# Minimal Structural Changes Determine Full and Partial Nicotinic Receptor Agonist Activity for Nicotine Analogues

**DOI:** 10.3390/molecules24152684

**Published:** 2019-07-24

**Authors:** Juan Pablo Gonzalez-Gutierrez, Martin Hodar, Franco Viscarra, Pablo Paillali, Nicolás Guerra-Díaz, Hernán Pessoa-Mahana, Juan José Hernández-Morantes, Horacio Pérez-Sánchez, Isabel Bermúdez, Miguel Reyes-Parada, Patricio Iturriaga-Vásquez

**Affiliations:** 1 Departamento de Química Orgánica y Fisicoquímica, Facultad de Ciencias Químicas y Farmacéuticas, Universidad de Chile, Santiago 8380494, Chile; 2 Departamento de Ciencias Químicas y Recursos Naturales, Facultad de Ingeniería y Ciencias, Universidad de la Frontera, Temuco 4811230, Chile; 3 Facultad de Enfermería, Universidad Católica de Murcia, 30107 Murcia, Spain; 4 Structural Bioinformatics and High Performance Computing Research Group (BIO-HPC), Universidad Católica de Murcia, 30107 Murcia, Spain; 5 School of Life sciences, Oxford-Brookes University, Oxford OX3 0BP, UK; 6 Centro de Investigación Biomédica y Aplicada (CIBAP), Escuela de Medicina, Facultad de Ciencias Médicas, Universidad de Santiago de Chile, Santiago 9170022, Chile; 7 Facultad de Ciencias de la Salud, Universidad Autónoma de Chile, Sede Talca 3467987, Chile

**Keywords:** α4β2 nAChR, pyrrolidine ethers, partial agonist, docking

## Abstract

Neuronal α4β2 nicotinic acetylcholine receptors (nAChRs) are ligand-gated ion channels (LGIC) that have been implicated in nicotine addiction, reward, cognition, pain disorders, anxiety, and depression. Nicotine has been widely used as a template for the synthesis of ligands that prefer α4β2 nAChRs subtypes. The most important therapeutic use for α4β2 nAChRs is as replacement therapy for smoking cessation and withdrawal and the most successful therapeutic ligands are partial agonists. In this case, we use the N-methylpyrrolidine moiety of nicotine to design and synthesize new α4β2 nicotinic derivatives, coupling the pyrrolidine moiety to an aromatic group by introducing an ether-bonded functionality. Meta-substituted phenolic derivatives were used for these goals. Radioligand binding assays were performed on clonal cell lines of hα4β2 nAChR and two electrode voltage-clamp experiments were used for functional assays. Molecular docking was performed in the open state of the nAChR in order to rationalize the agonist activity shown by our compounds.

## 1. Introduction

**Figure 1:**
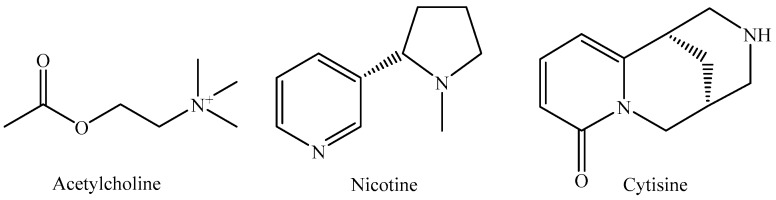
Classical nAChRs agonists.

Neuronal nicotinic acetylcholine receptors (nAChRs) are pentameric ligand-gated ion channels [1], expressed in the central and peripheral nervous systems that respond to the neurotransmitter acetylcholine (ACh) [2] and exogenous compounds such as nicotine and cytisine (Figure 1) [3,4]. Mammalian neuronal nAChRs assemble from combinations of nine α(α2 −α10) and three β(β2 − β4) subunits [3]. The α4β2 nAChR contains two α4β2 pairs and a fifth subunit that can be β2 or α4, which generates two alternate receptor isoforms known as the (α4β2)2β2 and the (α4β2)2α4 nAChRs [5].

The binding of an agonist induces the opening of the ion channel, which allows a flow of cations through the cell membrane, and, consequently, modulates the release of different neurotransmitters [6]. nAChR are involved in different CNS functions and disorders such as cognition, analgesia, nicotine addiction [7,8], anxiety, depression, and attention-deficit hyperactivity disorder [9] and constitute an established target for drugs aiding smoking cessation [10] such as varenicline [11].

The crystal structure of the ACh-binding protein (AChBP) from different species in the complex with several ligands, as well as derived homology models, have revealed the structural characteristics of the extracellular domain of nAChRs, as well as critical ligand-receptor interactions [12]. In addition, the recent description of the X-ray structure of a near-intact α4β2 nAChR bound to nicotine [13,14] has provided new insights regarding both the agonist binding site (the orthosteric site) and the structural requirements of its ligands. In the neuronal nAChRs, the orthosteric binding site is located at the interface formed between an α subunit (α3, α4, α7: the positive side) and a complementary subunit (α or β: the negative side) [15,16]. The ACh binding site, at the subunit interfaces, is formed by loops A, B, and C on the “principal face” and loops D, E, and F on the “complementary face” [17]. There are several highly conserved aromatic amino acid residues at this binding site in different nAChRs subtypes. It has been shown that a conserved tryptophan of loop B is a key residue required for ACh and other agonists binding, as it stabilizes the ligand binding mode through a π-cation interaction [18]. Conformational changes of the C-loop located at the entrance of the agonist binding site occur when a competitive agonist binds to the orthosteric site, which leads to coupling the ligand binding to the opening of the ion channel [19].

Partial agonists of α4β2 nAChRs such as cytisine or varenicline are currently used for smoking cessation therapy [20,21]. The rationale behind this type of treatment is that these compounds might help diminish withdrawal symptoms (acting as agonists), while reducing smoking satisfaction (acting as antagonists). Despite their success, there is evidence that these compounds induce significant side effects, which makes it necessary to develop novel drugs with better efficacy and safety profiles.

**Figure 2:**
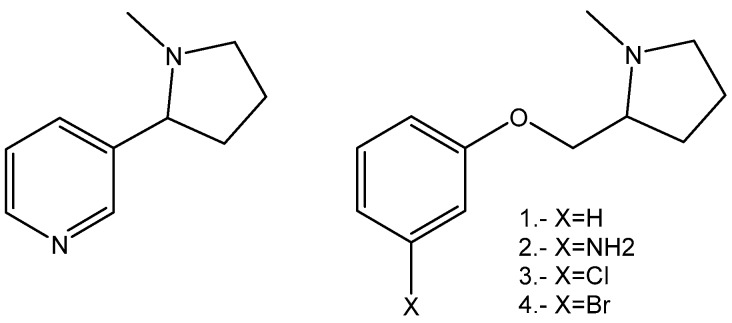
Pyrrolidine phenyl ether derivatives used in this work (right) and nicotine structure (left).

In this regard and based on previous theoretical, ethical, and experimental reports [22,23,24], we have designed and synthesized a small series of known and novel compounds, based on the nicotine structure, in order to modulate both their affinity and efficacy for α4β2 nAChRs. Thus, the pyridine moiety of nicotine was modified by moving the nitrogen atom outside of the ring, which either maintains the amino group or changes it by a halogen atom such as chloro or bromo. This substructure was linked to the N-methylpyrrolidine moiety of nicotine by an ether function (Figure 2).

Our design strategy considered that: (a) these molecules should still be able to establish the π-cation interaction, between the positively charged nitrogen of the N-methylpyrrolidine and the conserved aromatic residue W156 present at the ligand binding site of the α4β2 nAChRs [16,25], and (b) the aromatic moiety of our derivatives could interact with the aromatic cage into the binding site and could form hydrogen or halogen bridge(s).

## 2. Results and Discussion

### 2.1. Synthesis

**Figure 3:**
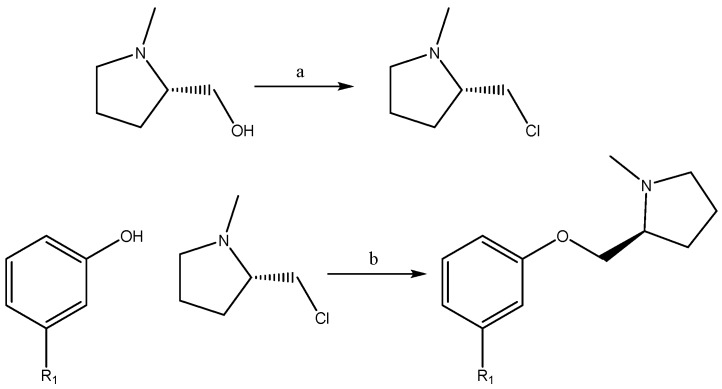
Synthesis Scheme: (a) SOCl_2_, THF-dry, reflux, 24 hours, (b) (S)-2-(chloromethyl)-1-methylpyrrolidine, phenolic derivatives, reflux 48 h, AcCN-dry.

For the synthesis of these phenylpyrrolidine ether derivatives, the phenolic derivatives, and (S)-(1-methylpyrrolidin-2-yl)methanol were obtained from usual commercial sources. As a first step, (S)-2-(chloromethyl)-1-methylpyrrolidine was synthesized using thionyl chloride as a halogenating agent in dry THF as solvent, at reflux for 24 hours under N_2_ atmosphere. After the reaction was complete, the solvent was evaporated on a rotary evaporator under reduced pressure. The (S)-2-(chloromethyl)-1-methylpyrrolidine obtained was used immediately. The phenolic derivatives were mixed with (S)-2-(chloromethyl)-1-methylpyrrolidine, in dry acetonitrile as solvent, and refluxed for 48 hours under N2 atmosphere (Figure 3). After the reaction was complete, the solvent was evaporated on a rotary evaporator under reduced pressure and the mixture of the reaction was purified using a combi-flash chromatograph. All compounds synthesized were obtained with a yield of 40% to 60% and the structures were confirmed by spectroscopy analysis.

In the specific case of the m-aminophenylpyrrolidine derivative, this was obtained from the reaction of m-nitrophenol and (S)-2-(chloromethyl)-1-methylpyrrolidine under the conditions already mentioned. The nitro group was then reduced via catalytic hydrogenation using 10% Pd/C as a catalyst in dry ethanol to give the corresponding aromatic amino group.

### 2.2. Biological Evaluation

#### 2.2.1. Competitive Radioligand Binding Studies

**Figure 4:**
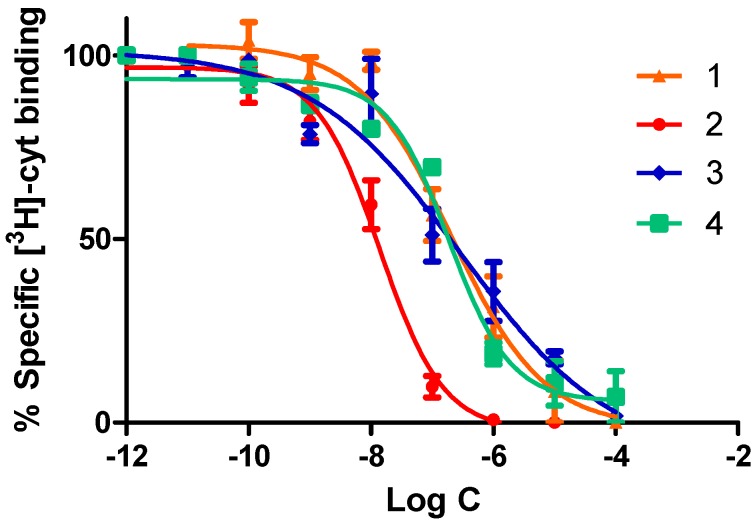
Effects of phenylpyrrolidine derivatives on binding of [^3^H]cytisine to human α4β2 nAChRs. Data points represent the mean ± SEM of four experiments. Each experiment was carried out in triplicate. The radioligand concentration in all displacement studies was 1 nM. The concentration-inhibition data was analyzed non-linearly.

First, we determined the half maximal inhibitory concentration (IC_50_) of the synthesized molecules for human α4β2 nAChR using [^3^H]-cytisine ([^3^H-Cyt]) as a radioligand. It is well known that [^3^H-Cyt] binds to the agonist (orthosteric) site of α4β2 nACRs [26]. The assays were carried out on membrane homogenates prepared from SH-EP-h α4β2 clonal cells, which have been shown to stably express human α4β2 nAChRs [27]. As shown in Figure 4, [^3^H]Cyt bound to α4β2 nAChRs was fully displaced by the synthesized compounds in a concentration-dependent and mono-phasic manner.

This pattern of radioligand binding inhibition indicates that the compounds bind to the agonist site of the receptors. Compounds tested showed a high affinity at α4β2 nAChRs, and, in all cases, [^3^H]Cyt was displaced with IC_50_ values in the nanomolar ranges. Compound 2 was the most potent with an IC_50_ about 15 times lower than those of compounds 1, 3, and 4, which showed similar affinities for the α4β2 nAChR (Table 1). These values are in agreement with the affinity previously reported for the compounds 1 and 3 [22].

As our results show, all compounds synthesized displaced [^3^H]Cyt from the agonist binding site of the α4β2 nAChR, which indicates that the ether function of our pyrrolidine derivatives does not affect their competitive binding properties. The inclusion of a substituent on the meta position of the aromatic group affected differentially the binding affinity depending on the substituent. Thus, the halogenated compounds (3 and 4) exhibited a similar affinity as compared to the unsubstituted derivative 1, whereas the introduction of an amino group at this position produced a clear increase of affinity. This effect has been reported with a hydroxyl analogue of compound 2, where the meta-hydroxyl position increases its affinity [23]. This might be due to the amino group, with donor/acceptor properties, being able to form hydrogen bridge(s) with some residues into the agonist binding site of the receptor.

#### 2.2.2. Functional Studies

**Figure 5:**
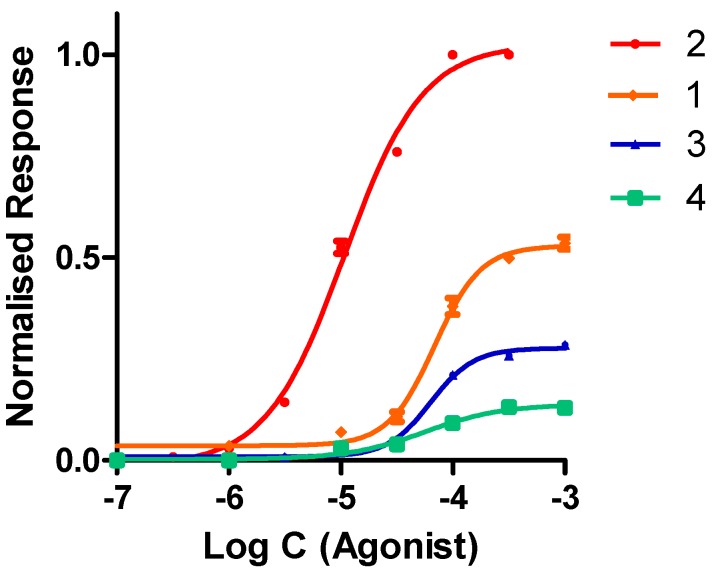
Interaction of phenylpyrrolidine derivatives with human α4β2 nicotinic receptors expressed in *Xenopus laevis* oocytes. Whole cell currents in response to agonist application were measured by a two-electrode voltage clamp. Concentration-response curves show activation of whole cell currents by compounds 1, 2, 3, and 4. Each point represents data from four to five cells, normalized to the amplitude of the maximal responses of ACh, which was stimulated by 1 mM of ACh. The data are fitted to a single component Hill curve.

The functional effects of our compounds were tested on α4β2 nAChR heterologously expressed in *Xenopus laevis* oocytes. Experiments were carried out by using the two-electrode voltage clamp procedure. The concentration-response data for our derivatives were best fitted to a single Hill equation (Figure 5). All compounds tested evoked inward currents in oocytes expressing α4β2 nAChR, which indicates that all of them are agonists. Nevertheless, the agonist properties of compounds showed differences both in potency and maximal responses (Table 1). The most potent agonist at the human α4β2 nicotinic receptor was compound 2, which was about seven-fold more potent than compound 1 (Table 1) and showed a potency similar to nicotine. The halogenated compounds 3 and 4 were equipotent as compared with compound 1. Compound 2 was a full agonist at human α4β2 nAChR (100% of efficacy). In contrast, compounds 1, 3, and 4 showed partial agonist properties at this receptor subtype (Table 1). Thus, the Imax of compound 1 was half of that of the full agonist ACh whereas the halogenated derivatives (compounds 3 and 4) were partial agonists with maximal responses that were, respectively, 29% and 13% of the ACh Imax. These results indicate that the introduction of a halogen atom at the meta position of our derivatives does not increase potency while dramatically and differentially decreasing the ability of the compounds to fully activate the receptors. In contrast, the presence of an amino group at this position increases affinity and potency and confers full agonist properties.

### 2.3. Molecular Docking Studies

**Figure 6:**
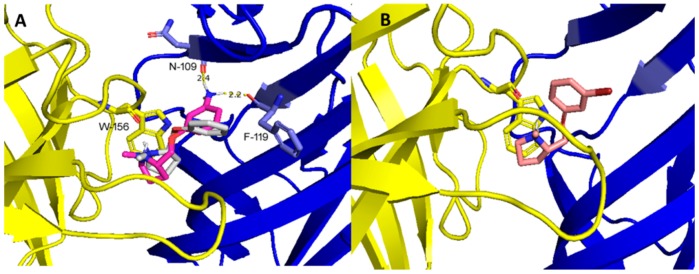
(**A**) Superimposed structures of compounds 1 (grey), and 2 (magenta) docked into the agonist binding site of the α4β2 nAChR. Compound 2 shows the additional interaction with β2N109 and β2F119. (**B**) Binding mode of compound 4 (pink) into the binding site of the α4β2 nAChR. The images show the π-cation interaction between the ligands and the α4W156 in the opened channel state conformation (C loop closed).

Binding studies indicated that our phenylpyrrolidine derivatives bind at the agonist (orthosteric) binding site of α4β2 nAChRs. Therefore, in an attempt to rationalize the differences in binding affinity, functional potency, and maximal responses of our derivatives, the possible binding modes of these compounds were analyzed by docking simulations on the α4β2 nAChR agonist binding site. The extracellular N-terminal ligand binding domain of the receptor was obtained from the X-ray structure of the human α4β2 nAChR (Protein Data Bank code 5KXI) [13] and used for the docking studies. Phenylpyrrolidine derivatives were docked into the α4(+)/β2(−) interface centering the grid on α4W156. This residue is equivalent to α1W149 on the muscle nAChR, which forms a π-cation interaction with the quaternary ammonium group of ACh and the protonated nitrogen of several agonists [30]. Docking results indicate that small changes in the nicotine structure, such as the addition of an ether linker between the pyrrolidine moiety and the aromatic part of the parent compound, does not generate unfavorable interactions within the binding site. However, the introduction of different substituents at the meta position of the aromatic moiety produces important changes for the binding modes. Thus, the halogenated derivatives (compounds 3 and 4) docked into the agonist binding site in a position allows establishing the classical π-cation interaction with the tryptophan (α4W156), and, at the same time, generates an unfavorable (electronic repulsive) interaction between the bulky halogen atom and the C-loop (Figure 6B). This binding mode correlates with the low potency and efficacy of these derivatives. Additionally, the unsubstituted compound 1 shows the same binding mode of the halogenated derivatives, but the absence of a bulky substituent into the aromatic group (i.e., chloro or bromo atoms) allows the C-loop to keep closed. As compounds 1, 3, and 4 exhibit similar affinities, the differences in the binding mode revealed by docking simulations might be related with the higher agonist efficacy of 1 as compared with the halogenated derivatives. On the other hand, the meta-amino derivative (compound 2) adopted a binding mode in which unfavorable interactions were not detected, and that, besides the π-cation interaction, exhibited additional hydrogen bonding between the drug amino group and the carbonyl group of asparagine (β2N109) and phenylalanine (β2F119) residues on the complementary β2 subunit (Figure 6A). These results align with the nicotinic profile of compound 2, which shows high binding affinity, considerable agonist potency, and full agonism.

## 3. Materials and Methods

### 3.1. Clonal Cell Lines

Membrane homogenates for [^3^H]-Cyt binding studies were prepared from the SH-EP-h α4β2 clonal cell line, which express human α4β2 nAChRs. Cells were maintained in Dulbecco’s modified Eagle’s medium (Invitrogen, UK) supplemented with 5% fetal serum, 10% horse serum, 2 mM l-glutamine, 10 IU/mL penicillin, 10 μg/mL streptomycin, 2.5 μg/mL amphotericin-B, 0.25 mg/mL zeocin, and 0.13 mg/mL hygromycin.

### 3.2. Ligand Binding Assays

Competition binding studies were performed on membrane homogenates prepared SH-EP-hα4β2 clonal cell line, using [^3^H]cyt (PerkinElmer, UK), as previously described [28]. Membrane homogenates were incubated at a final protein concentration of 30 to 50 µg per assay tube in a final volume of 250 µL of binding saline (in nM: 120 NaCl, 5 KCl, 1 MgCl2, 2.5 CaCl2, 50 Tris, pH 7.0) for 75 min at 4 °C with 1 nM [^3^H]cyt. For this binding assay, 10 µM nicotine was used to define nonspecific binding. Bound and free fractions were separated by rapid filtration through Whatman GF/C filters presoaked in binding saline supplemented with 0.1% polyethyleneimine. Radioactivity was quantified by liquid scintillation spectrometry.

### 3.3. Nicotinic Acetylcholine Receptors Expression in Xenopus laevis Oocytes

The α4β2 nAChRs (wild type) were expressed heterologously in defolliculated oocytes from *Xenopus laevis*, which were dissected from adult female *X. laevis* frogs (Nasco, Fort Atkinson, WI, USA). The Oxford Brookes University Animal Research Committee, in accordance with the guidelines of the 1986 Scientific Procedures Act of the United Kingdom, approved the care and use of *X. laevis* frogs in this study. A mixture of 1:1 of human of α4 and β2 subunits for α4β2 subtype cDNAs were injected into the nuclei of oocytes in a volume of 23 nL/oocyte by using a Nanoject Automatic Oocyte Injector (Drummond Scientific, Broomall, PA, USA). After injection, oocytes were incubated at 17 °C in OPS (NaCl, KCl, CaCl2, MgCl2, pH 7.4) supplemented with a mixture of penicillin-streptomycin-anphotericin-B (10,000 IU/mL penicillin, 10 mg/mL streptomycin, and 25 µg amphotericin-B/mL) and amikacin (100 µg/mL). Experiments were performed on oocytes 2 to 6 days after injection.

### 3.4. Electrophysiological Recordings

Electrophysiological recording from oocyte post-injection was made at room temperature using a standard two electrode voltage clamp technique with an automatic multichannel system (HiClamp, Multichannel Systems, Reutlingen, Germany). Oocytes were impaled by two borosilicate capillary glass (Harvard Instrument: 150 TF GC) microelectrodes filled with 3 M KCl (0.3–2.0 MΩ) and voltage-clamped at −60 mV. During recording, oocytes were perfused with OPS (oocytes perfusion solution), as described in the manual of HiClamp. Agonist responses were normalized to the amplitude of the responses induced by 1 mM ACh, which is the maximal ACh concentration at α4β2 nAChRs. Between each successive ACh or compound application, the cell was perfused with OPS solution for 3 min to allow drug clearance and prevent receptor desensitization.

### 3.5. Molecular Docking Studies

Molecular docking of the phenylpyrrolidine derivatives at the agonist binding domain of the α4β2 (PDB code 5KXI; [13]) was investigated using the Lamarckian genetic algorithm search method using software AutoDock v4.2. The receptor was kept rigid, while full flexibility was allowed for the ligands to translate/rotate. Polar hydrogens were added to the receptors and Kollman-united atom partial charges along with atomic solvation parameters, which were assigned to the individual protein atoms. The three-dimensional structures of each ligand were generated using the SPARTAN’11 program and were then their energy was minimized. For each ligand, a rigid root and rotatable bonds were assigned automatically. The non-polar hydrogens were removed and the partial charges from these were added to the carbons (Gasteiger charges). The atom type for aromatic carbons was reassigned in order to use the AutoDock v4.2 aromatic carbon grid map. Docking was carried out using 60 × 60 × 60 grid points with a default spacing of 0.375 Å. The grid was positioned to include the full ligand binding pocket in the central part of the α4β2 subunit interfaces in order to allow extensive sampling around residue α4W156. Within this grid, the Lamarckian genetic search algorithm was used and calculated using 100 different runs (i.e., 100 dockings).

## 4. Chemistry

NMR spectra were recorded using Bruker AMX 400 spectrometers. Chemical shifts are reported relative to TMS (δ = 0.00) and coupling constants (*J*) are given in Hz. IR spectra were recorded using Cary 630 FTIR-ATR (Agilent, Santa Clara, USA). The elemental analyses for C, H, and N were performed on a CE Instruments (model EA 1108) analyzer (Waltham, MA, USA). Reactions and product mixtures were routinely monitored by thin-layer chromatography (TLC) on silica gel-pre-coated F254 Merck plates (Darmstadt, Germany), using a mixture of CH_3_Cl/CH_3_OH as a mobile phase. All reagents and solvents were commercially available and were used without further purification.

### 4.1. General Procedures

#### 4.1.1. Synthesis of (S)-2-(chloromethyl)-1-Methylpyrrolidine

(S)-(1-methylpyrrolidin-2-yl) metanol were previously transformed in their corresponding (S)-2-(chloromethyl)-1-methylpyrrolidine, using thionyl chloride in dry THF as a solvent and refluxed under a nitrogen atmosphere. The mixture was maintained at room temperature for 24 h, and then the solvent, excess reagents, and remaining HCl and SO_2_ were evaporated under vacuum. The (S)-2-(chloromethyl)-1-methylpyrrolidine was used immediately for the next reaction.

#### 4.1.2. Synthesis of (S)-1-methyl-2-(phenoxymethyl)pyrrolidine Derivatives

(S)-3-(chloromethyl)-1-methylpyrrolidine was dissolved in 50 mL of dry acetonitrile stirred at room temperature. One equivalent of phenol derivatives in 30 mL of dry acetonitrile was added drop by drop. The reaction mixture was kept at reflux with constant stirring for 48 h. Then, the solvent was evaporated and the mixture was dissolved in water, adjusted to pH 8.0, extracted with CH_3_Cl, and purified by chromatography using a combi flash chromatograph. The hydrochloride salt was obtained from acetone.

### 4.2. (S)-1-methyl-2-(phenoxymethyl)pyrrolidine Ether (1)

Obtained as a light yellow oil in 56% yield, salt m.p. 175–178 °C.^1^H-RMN(DMSO-d_6_) δ: 8.08 (d, J = 7.8 Hz, 2H, 2-H y 6-H), 7.75 (t, J = 7.5 Hz, 1H, 4-H), 7.59 (t, J = 7.7 Hz, 2H, 3-H y 5-H), 4.76 (d, J = 2.9 Hz, 1H, 1′-H), 4.58 (dd, J = 13.1, J = 6.7 Hz, 1H, 1′-H), 3.95 (s, 1H, 2′-H), 3.78 (s, 1H, 3′-H), 3.29 (s, 1H, 3′-H), 3.08 (s, 3H, 6′-H), 2.45 (m, 1H, 5′-Ha), 2.26 (m, 1H, 5′-Hb), 2.12 (ddd, J = 31.3, 14.5, 7.1 Hz, 2H, 4′-Ha y 4′-Hb). ^13^C-RMN (DMSO-d_6_) δ: 165.3 (1-C), 133.6 (4-C), 129.4 (2-C y 6-C), 129.1 (3-C), 128.7 (5-C), 66.0 (2′-C), 62.5 (1′-C), 56.1 (3′-C), 39.5 (6′-C), 26.6 (5′-C), 21.7 (4′-C). IR (cm^−1^) 3032, 2978, 1279, 1112 [22].

### 4.3. (S)-1-methyl-2-(3′-aminophenoxymethyl)pyrrolidine Ether (2)

Obtained as a light brown oil in 41% yield, salt m.p. 198–200 °C. ^1^H-RMN (DMSO-d_6_) δ: 7.90 (s, 1H, 2-H), 7.79 (d, J = 7.5 Hz, 1H, 6-H), 7.56 (dd, J = 13.3, 7.8 Hz, 1H, 4-H), 7.48 (t, J = 8.5 Hz, 1H, 5-H), 4.63 (s, 2H, 1′-H), 3.81 (s, 1H, 2′-H), 3.56 (s, 1H, 3′-Ha), 3.13 (s, 1H, 3′-Hb), 2.87 (s, 3H, 6′-H), 2.24 (dt, J = 13.8, 6.9 Hz, 1H, 5′-Ha), 1.99 (m, 2H, 5′-Hb y 4′-Ha), 1.87 (dt, J = 20.7, 7.6 Hz, 1H, 4′-Hb). ^13^C-RMN (DMSO-d_6_) δ: 167.9 (1-C), 162.6 (3-C), 132.4 (5-C), 127.17 (6-C), 122.7 (4-C), 117.8 (2-C), 68.9 (2′-C), 64.2 (1′-C), 58.8 (3′-C), 42.2 (6′-C), 27.7 (5′-C), 23.6 (4′-C). IR (cm^−1^) 3442, 3360, 3046, 2978, 1285, 1270, 1115. Anal. Calcd for C_12_H_19_ClN_2_O: C, 59.38; H, 7.89; N, 11.54. Found: C, 58.74; H, 7.72; N, 11.46.

### 4.4. (S)-1-methyl-2-(3′-Chlorophenoxymethyl)pyrrolidine Ether (3)

Obtained as a light yellow oil in 61% yield, salt m.p. 158–160 °C. ^1^H-RMN (DMSO-d_6_) δ: 8.02 (s, 1H, 2-H), 7.77 (d, J = 7.8 Hz, 1H, 6-H), 7.59 (t, J = 7.8 Hz, 1H, 5-H), 7.45 (dd, J = 11.3, 6.9 Hz, 1H, 4-H), 4.63 (s, 2H, 1′-H) 3.80 (s, 1H, 2′-H), 3.58 (s, 1H, 3′-H), 3.09 (d, J = 9.0 Hz, 1H, 3′-H), 2.83 (s, 3H, 6′-H), 2.25 (m, 1H, 5′-H), 2.05 (m, 1H, 5′-H), 1.92 (m, 2H, 4′-H ). ^13^C-RMN (DMSO-d_6_) δ: 166.8 (1-C), 135.3 (2-C), 135.2 (4-C), 135.2 (3-C), 131.9 (5-C), 129.40 (6-C), 70.80 (2′-C), 59.74 (1′-C), 57.83 (3′-C), 41.33 (6′-C), 26.98 (5′-C), 22.95 (4′-C). IR (cm^-1^) 3075, 2995, 1277, 1108, 740 [22].

### 4.5. (S)-1-methyl-2-(3′-bromophenoxymethyl)pyrrolidine Ether (4)

Obtained as a light yellow oil in 52% yield, salt m.p. 147–1149 °C. ^1^H-RMN (DMSO-d_6_) δ: 8.03 (s, 1H, 2-H), 7.93 (d, J = 7.7 Hz, 1H, 6-H), 7.82 (d, J = 7.9 Hz, 1H, 4-H), 7.47 (t, J = 7.8 Hz 1H, 5-H), 4.60 (dt, J = 12.7, 9.6 Hz, 2H, 1′-H), 3.82 (s, 1H, 2′-H), 3.56 (s, 1H, 3′-Ha), 3.14 (s, 1H, 3′-Hb), 2.90 (s, 3H, 6′-H), 2.23 (m, 1H, 5′-H), 2.03 (m, 1H, 5′-H), 1.90 (m, 2H, 4′-Ha y 4′-Hb). ^13^C-RMN (DMSO-d_6_) δ: 165.2 (1-C), 135.9 (4-C), 131.2 (5-C), 129.5 (2-C), 127.3 (6-C), 121.0 (3-C), 66.4 (2′-C), 61.5 (2′-C), 56.2 (3′-C), 39.6 (6′-C), 25.1 (5′-C), 20.9 (4′-C). IR (cm^−1^) 2980, 1281, 1115, 686. Anal. Calcd for C_12_H_17_BrClNO: C, 47.01; H, 5.59; N, 4.57. Found: C, 46.69; H, 5.25; N, 4.15.

## 5. Conclusions

Our results indicate that phenylpyrrolidine ether derivatives bind to the orthosteric site of the α4β2 nAChR. Binding experiments show that all compounds synthesized fully displaced [3H]cyt from its binding site with IC_50_s in the nanomolar range using the m-amino derivative (compound 2), which shows the best affinity (IC_50_ =13 nM). Functional studies show that the introduction of a halogen atom into the meta position of the aromatic ring dramatically and differentially decreases efficacy for the agonist activity as compared with compounds 1 (the unsubstituted derivative) and 2 (the meta-amino derivative). In addition, the meta-amino derivative was the most potent (EC_50_ =10 μM) agonist in this series, was equipotent to nicotine, and was a full agonist. Notably, a whole range of efficacies could be detected in this small series of compounds. Molecular docking studies indicate that differential affinities, potencies, and efficacies of the compounds tested, might be related with their different binding modes (yielding different interactions) at the agonist binding site of the α4β2 nAChR. Thus, our data suggest some amino acid residues (and their corresponding three dimensional assemblies) at the binding site, whose role, particularly in the modulation of efficacy of agonists, could be explored.

The ample spectrum of efficacies exhibited by this small series of compounds supports our idea that subtle structural modifications can drastically modulate the nicotinic profile of agonists. It also highlights the relevance of searching for novel compounds with varied pharmacodynamics, which might be useful for treating patients with diverse characteristics. Further experiments, including additional compounds, are necessary to confirm the hydrogen bonding effect and to evaluate the therapeutic potential of the compounds described, particularly in the case of the partial agonists.

## Figures and Tables

**Table 1:** Binding affinity (IC_50_), potency (EC_50_), and maximal current (Imax) of phenylpyrrolidine derivatives at human α4β2 nAChR. In the case of affinity (IC_50_) determinations, data represent the mean ± SEM of four experiments. Each experiment was done in triplicate. Assays were carried out on membrane homogenates prepared from SH-EP-hα4β2 clonal cells and the radioligand concentration in all displacement studies was 1 nM. In the case of potency (EC_50_) and maximal current responses (Imax) determinations, values represent the mean ± SEM of four to five independent experiments. Assays were carried out on human α4β2 nAChR expressed heterologously in *Xenopus laevis* oocytes. IC_50_ estimates for nicotine determined previously under the same conditions [28,29] were included for comparative purposes.

Compound	α4β2 IC_50_ (nM)	α4β2 EC_50_ (μM)	Imax
Nicotine	1.2	10	1.00
1	204.0 ± 6.0	68.8 ± 1.3	0.54 ± 0.01
2	13.8 ± 0.5	10.8 ± 0.7	1.00 ± 0.01
3	221.0 ± 10.0	63.3 ± 2.1	0.29 ± 0.01
4	188.0 ± 7.0	56.7 ± 1.7	0.13 ± 0.01
